# Acute leukoencephalopathy associated with daratumumab treatment in POEMS syndrome: a case report

**DOI:** 10.3389/fimmu.2024.1451693

**Published:** 2024-09-26

**Authors:** Lukas Steinegger, Nathalie Nierobisch, Anthony De Vere-Tyndall, Bettina Schreiner, Patrick Roth, Ludwig Kappos, Veronika Kana, Marina Herwerth

**Affiliations:** ^1^ Department of Neurology, University Hospital Zurich, Zurich, Switzerland; ^2^ Neuroscience Center Zurich, University of Zurich and ETH Zurich, Zurich, Switzerland; ^3^ Department of Diagnostic and Interventional Neuroradiology, University Hospital Zurich, Zurich, Switzerland; ^4^ Institute of Experimental Immunology, University of Zurich, Zurich, Switzerland; ^5^ Research Center for Clinical Neuroimmunology and Neuroscience Basel (RC2NB), University Hospital and University of Basel, Basel, Switzerland; ^6^ Department of Neurology, Klinikum rechts der Isar, Technische Universität München, Munich, Germany; ^7^ Institute of Pharmacology and Toxicology, University of Zurich, Zurich, Switzerland

**Keywords:** POEMS, daratumumab, rapidly progressive leukoencephalopathy, CD38-antibody, toxic leukoencephalopathy

## Abstract

**Objectives:**

Daratumumab, a monoclonal antibody against CD38, is increasingly used in the treatment of multiple myeloma, other hematological malignancies and autoimmune diseases. Little is known about its CNS toxicity. We present a case of a patient with POEMS syndrome (syndrome of polyneuropathy, organomegaly, endocrinopathy, monoclonal gammopathy and skin changes) who developed an acute leukoencephalopathy shortly after initiation of therapy with daratumumab.

**Methods:**

Case report following the CARE case report guidelines

**Results:**

The patient presented with symptoms of headache and diffuse worsening of a pre-existing tetraparesis. MRI showed a rapidly progressive leukoencephalopathy. Extensive diagnostic evaluation revealed no specific cause, suggesting the leukoencephalopathy to be caused by daratumumab.

**Discussion:**

Our report highlights a probably rare, but clinically significant adverse effect of daratumumab and underlines the necessity of raised vigilance for neurological side effects in patients treated with daratumumab.

## Introduction

Daratumumab is a fully human IgG1κ monoclonal antibody directed against CD38 (1). CD38 is expressed in several immune cells such as B-, T- and NK- and myeloid progenitor cells (1), but also in cells of the central nervous system, including astrocytes, oligodendrocytes and pyramidal neurons (2, 3). The main indications for daratumumab, that is usually combined with immunomodulators such as lenalidomide and steroids, are multiple myeloma and other hematological malignancies (1). Accumulating evidence suggests an efficacy also in patients with POEMS syndrome, a rare paraneoplastic syndrome typically occurring in the context of a monoclonal gammopathy with production of lambda light chains (4, 5). Furthermore, daratumumab is increasingly used to treat autoimmune diseases (e.g. autoimmune encephalitis) with the goal to remove autoantibody-producing plasma cells (6).

Common side effects of daratumumab include infusion reactions, bone marrow suppressive effects and infectious complications. Neurological side effects are not commonly reported (1, 6). However, recent studies described patients with multiple myeloma suffering from leukoencephalopathy during treatment with daratumumab (7, 8). Here, we present the case of a 60-year-old woman with POEMS syndrome who developed an acute, rapidly progressive leukoencephalopathy shortly after initiation of treatment with daratumumab.

## Case report

The 61-year-old female patient initially presented in our outpatient clinic with a progressive demyelinating polyneuropathy, manifesting with a distally symmetric sensorimotor tetraparesis and extremity ataxia. Further diagnostic work up revealed a monoclonal gammopathy type IgA lambda, elevated serum levels of vascular endothelial growth factor (VEGF) and extravascular volume overload (bilateral pleural effusions), which led to the diagnosis of a POEMS syndrome according to the diagnostic criteria (4). A therapy with daratumumab, lenalidomide and dexamethasone was started. Under this therapy, the patient reported a slow but steady improvement of her polyneuropathy symptoms. Follow-up nerve conduction studies revealed a clear improvement of the known demyelinating polyneuropathy compared to the prior examination before treatment initiation with daratumumab.

Two months after initiation of the therapy, she noticed a new-onset headache with increasing intensity over the following days, accompanied by a worsening of the preexisting neurologic deficits. Clinical examination showed a symmetrical, distally predominant, sensorimotor tetraparesis with left-predominant ataxia, comparable to the degree before initiation of daratumumab. While the patient had a generalized areflexia at the prior visit, we noted ameliorated deep-tendon reflexes. Cranial nerves and mental status examination were normal. General examination was normal except for an elevated blood pressure (initially 210/82 mmHg) with spontaneous normalization within hours. We admitted the patient to our neurology in-patient ward.

**Figure 1:**
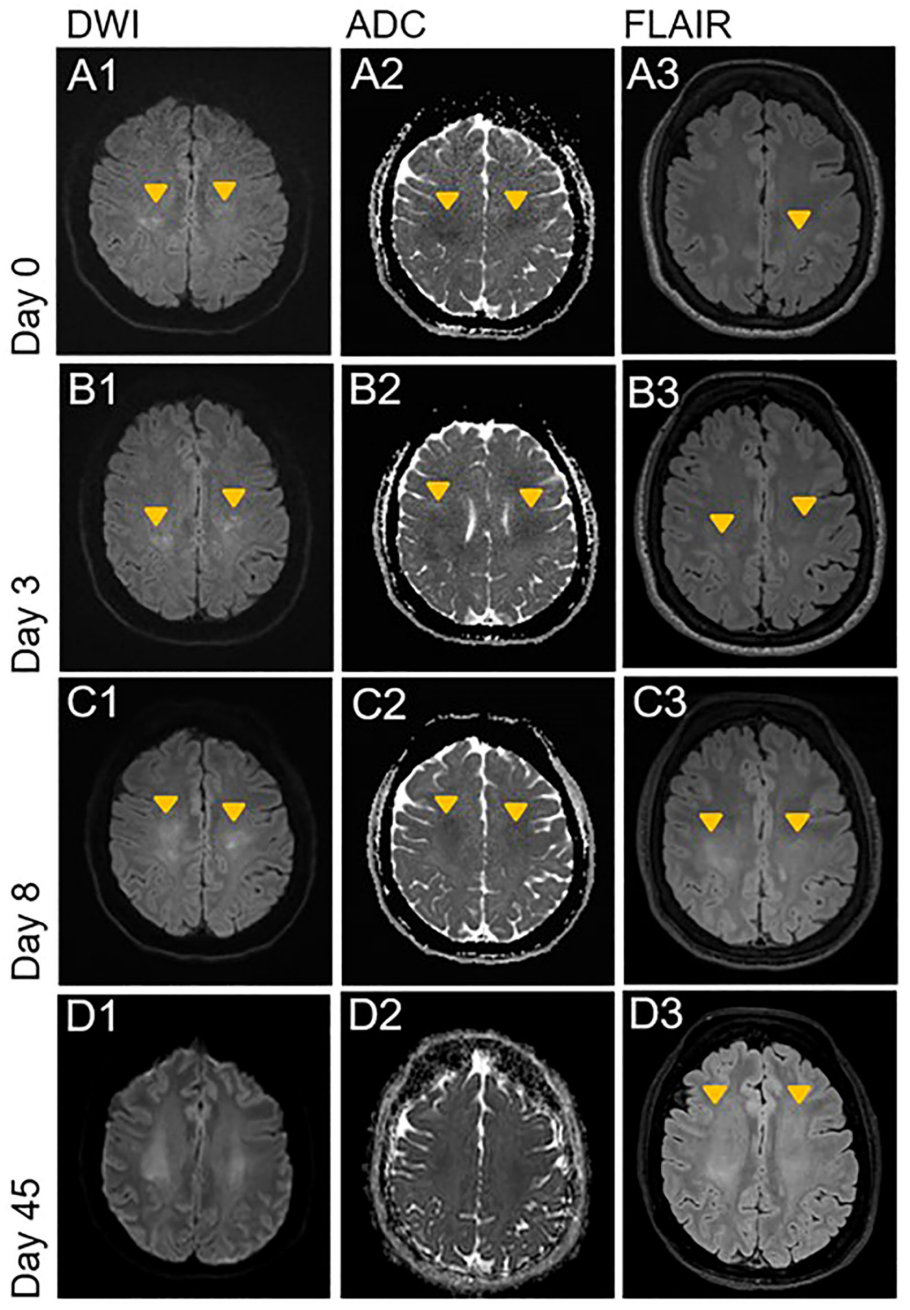
Acute progressive leukoencephalopathy shortly after initiation of therapy with daratumumab. DWI, ADC and FLAIR sequences of the initial brain MRI examination on day 0 and follow up imaging on day 3, 8 and 45. Orange arrowheads indicate new or progressive lesions. **(A1-A3)** Bilateral, diffuse diffusion restriction in the deep white matter of the parietal centrum semiovale with discrete FLAIR hyperintense correlate on left parietal side. **(B1-C3)** Progression of the bilateral and diffuse diffusion restriction with T2w and FLAIR hyperintense correlate on the left parietal side. New correlating FLAIR hyperintensities on the right parietal side. Start of pseudo-normalization of ADC in the more parietal areas **(C2)**. **(D1-D3)** Further progression of the bilateral confluent FLAIR hyperintensities to the frontal white matter of centrum semiovale. Almost complete pseudonormalization of ADC with residual diffusion restriction. FLAIR, Fluid attenuated inversion recovery; DWI, Diffusion weighted imaging; ADC, Apparent diffusion coefficient.

MRI of the brain on the following day showed bilateral, diffuse hyperintense lesions on diffusion-weighted imaging (DWI) sequences with low apparent diffusion coefficient (ADC) values in the white matter of both hemispheres (**Figure 1**, Images A1-A2), with a discrete correlate on fluid attenuated inversion recovery (FLAIR-)/T2-weighted sequences in the left parietal lobe (**Figure 1**, Image A3). Contrast enhancement was not detectable (not shown). Follow up imaging three and eight days later showed progression of the lesions (**Figure 1**, Images B1-B2 and C1-C2), now with a clear correlate on FLAIR/T2-weighted sequences (**Figure 1**, Images B3 and C3). Additional imaging with vessel-wall sequences showed no signs of pathological contrast enhancement of the intracerebral vessels.

Routine laboratory tests were unremarkable (normal full blood count with leukogram, erythrocyte sedimentation rate, coagulation testing and blood chemistry tests including C-reactive protein). Cerebrospinal fluid (CSF) analysis was normal (one cell/µl, normal protein levels). CSF-specific oligoclonal bands were not detectable. Further CSF cytological analysis and flow cytometry showed normal results. Viral PCR for JC virus was negative. Extensive testing for antibodies associated with autoimmune encephalitis and onconeural antibodies in our own and in an external reference laboratory was negative. Screening for systemic vasculitis and collagenosis was unrevealing (including testing for anti-nuclear antibodies, anti-neutrophil cytoplasmic antibodies (ANCA) including MPO- and PR3-ANCA, rheumatoid factor, anti-phospholipid antibodies).

Because of the temporal association with the daratumumab treatment, we assumed a toxic-metabolic leukoencephalopathy and stopped therapy with daratumumab. In view of the improvement of muscle strength and resolution of headache after stopping daratumumab, we refrained from empiric therapy with steroids. Despite clinical improvement, a follow-up MRI six weeks later showed a progression of the diffuse T2/FLAIR-hyperintensities (**Figure 1**, Images D1-D3). Another follow-up MRI four months later showed stable white matter lesions without any signs of radiologic activity. At the last visit five months later, the patient remained clinically stable without laboratory signs of progression of the POEMS syndrome, including stable levels of VEGF and free lambda light-chains.

## Discussion

Despite increasing clinical use of daratumumab, data about neurological side effects is sparse. In the literature, we found four reported cases of leukoencephalopathy after starting a therapy with daratumumab in patients with multiple myeloma (7–9). One patient suffered from a rapidly progressive bilateral weakness and numbness, occurring one month after initiation of therapy with daratumumab, bortezomib, pomalidomide and dexamethasone. MRI changes were very similar to the findings in our patient. Symptoms rapidly improved after treatment with glucocorticoids and stopping daratumumab, however MRI changes persisted over several months despite clinical improvement (8). Another patient suffered from an acute alteration of consciousness leading to coma immediately after the first infusion of daratumumab. MRI revealed diffuse bihemispheric and cerebellar T2-hyperintense lesions with some correlate on DWI. This patient also improved rapidly after administration of steroids (9). The two other patients had a more subacute presentation starting several months after initiation of daratumumab in combination with an immunomodulator and dexamethasone. Both patients suffered from progressive cognitive deficits and pyramidal signs with lethal outcome in one of the patients and progressive neurological deficits despite stopping treatment with daratumumab in the other (7). To our knowledge, no cases of leukoencephalopathy under daratumumab therapy have been reported in POEMS syndrome so far. In addition, we found no reports about leukoencephalopathy under other monoclonal antibodies against CD38.

The temporal association of the treatment with daratumumab and the rapidly progressive leukoencephalopathy, the lack of a better explanation and the similar observations in previous case reports (7–9) suggest a causal relationship between daratumumab and the leukoencephalopathy. Given that daratumumab crosses the blood-brain-barrier (10), it is reasonable to assume that toxicity is related to binding on CD38 expressing cells of the central nervous system (CNS), such as astrocytes and neurons (1–3). Another possible explanation for the leukoencephalopathy might have been a direct CNS manifestation of the POEMS syndrome. A recent study analyzing brain MRI scans of 41 patients with POEMS syndrome showed frequent meningeal thickening and vascular white matter changes, but no leukoencephalopathy similar to that observed in our patient (11). Some studies also indicate that patients with POEMS syndrome are at higher risk for ischemic strokes, possibly due to high levels of VEGF and other cytokines leading to a cerebral vasculopathy (12, 13). However, our patient showed no signs of vasculopathy in MRI vessel-wall imaging. The diffuse DWI lesions did not respect vascular territories and progressed over two weeks. Both features are unusual for an ischemic etiology. Moreover, the clinical, electrophysiological and laboratory improvement of the POEMS syndrome indicated a good response to the treatment with daratumumab. Therefore, it is rather unlikely that the patient suffered from a new onset CNS manifestation of POEMS. Posterior reversible encephalopathy syndrome (PRES), a disorder of reversible subcortical vasogenic brain edema with a predominance in the parieto-occipital regions (14), could hypothetically also be the reason for the findings in our patient. The blood pressure fluctuations and possibly the administration of daratumumab itself could serve as a trigger for a PRES. The predominantly cytotoxic, not vasogenic edema and the atypical distribution of the MRI changes argues against PRES as the cause for the persistent leukoencephalopathy of our patient. The similarity of radiological findings with MRI patterns generally found in acute toxic leukoencephalopathies of various cause further speaks in favor of a toxic cause (15).

In summary, the presented case, describing an acute onset of bihemispheric leukoencephalopathy after therapy initiation with daratumumab in a patient with POEMS syndrome, strongly suggests a causal relation of this apparently rare complication of daratumumab treatment. Considering its emerging use in an expanding number of indications, we suggest raised vigilance for neurological symptoms. Systematic clinical and radiological monitoring of CNS side effects is needed to understand the real frequency and possible causal mechanisms of daratumumab-associated leukoencephalopathy better.

## Data Availability

The original contributions presented in the study are included in the article/supplementary material. Further inquiries can be directed to the corresponding authors.
